# Evaluating the potential of using urine and saliva specimens for malaria diagnosis in suspected patients in Ghana

**DOI:** 10.1186/s12936-020-03427-x

**Published:** 2020-09-29

**Authors:** Enoch Aninagyei, Joseph Abraham, Paul Atiiga, Shadrach Duodu Antwi, Stephen Bamfo, Desmond Omane Acheampong

**Affiliations:** 1 Department of Biomedical Sciences, School of Basic and Biomedical Sciences, University of Health and Allied Sciences, PMB 31, Ho, Volta Region Ghana; 2 Department of Biomedical Sciences, School of Allied Health Sciences, University of Cape Coast, Cape Coast, Ghana

**Keywords:** Malaria antigens in urine, Malaria antigens in saliva, Ga west municipality, Ghana

## Abstract

**Background:**

This study aimed at detecting PfHRP2 and pLDH malaria antigens in urine and salivary specimens of suspected malaria patients using RDT kits, and identifying factors influencing the detection of these antigens.

**Methods:**

Malaria rapid test kit (SD Bioline RDT kit) was used to detect malaria antigens, PfHRP2 and pLDH, in blood, urine and saliva samples received from patients suspected of malaria. Subsequently, malaria parasitaemia was determined. From the same patients, body temperature readings and haemoglobin concentrations were recorded. Also, micro-haematuria and saliva occult blood were determined. Relative to blood, the sensitivities and the performance of urine and saliva as alternative samples were evaluated.

**Results:**

A total of 706 suspected malaria patients provided all three specimens. Prevalence of malaria by microscopy and RDT was 44.2% and 53.9%, respectively. Compared to blood, the sensitivities of urine and saliva were 35.2% and 57.0% respectively. Haemoglobin concentration < 9.9 g/dL, body temperature > 38.7 °C and occult blood influenced the detection of malaria antigens in both urine and saliva. Furthermore, the antigens were not detected in urine and saliva when parasitaemia was < 60,000 parasites/µL and < 40,000 parasites/µL, respectively.

**Conclusion:**

Saliva, with or without blood contamination, was found to be more efficient that urine samples. Therefore these non-blood specimens have the potential to be used as non-invasive samples for malaria diagnosis. However, this approach is useful in severe to moderate anaemia, hyperthermia, parasitaemia > 60,000 parasites/µL and samples contaminated with blood.

## Background

Malaria is one of the highest killer diseases predominant in sub-Saharan Africa (SSA). In 2018, about 212 million new infections and 381,000 deaths were reported in SSA [1]. In Ghana, *Plasmodium falciparum* account for 90–98% of all malaria cases [2]. The World Health Organization (WHO) recommends laboratory confirmation of all malaria cases before initiation of treatment [3]. Timely diagnosis and appropriate treatment are essential for addressing the global burden of malaria. Blood specimen is commonly used to perform malaria diagnosis using microscopes and rapid diagnostic test (RDTs) kits. Blood collection requires an invasive procedure using hypodermic needles [4].

Invasive phlebotomy can cause adverse effects, such as pain or bruising at the site of puncture, fainting, nerve damage and haematoma [5] in patients. Again, poor infection-control practices can lead to microbial infections at the site where the needle was inserted into the skin [6] and for that matter, both patients and health workers can be exposed to blood borne infections from infected people [7–9].

Due to the potential demerits of invasive phlebotomy, diagnosis of diseases based on non-invasive procedures have been suggested and evaluated in some studies [10]. Urine and saliva are the two most popular alternatives to blood for diagnosis of diseases such as malaria [11]. Collection of urine and saliva do not require invasive procedures. It is simple, safe, painless and can be done by individuals with limited training, including patients themselves. No special equipment is also needed for collection, and it allows for multiple or serial collections outside of the hospital.

Previous studies in Ghana and in the Philippines detected PfHRP2 antigens in saliva of malaria patients using enzyme immuno-assays [12, 13]. Using RDT kits, sensitivities of pLDH was found to be 77.9% in whole saliva and 48.4% in saliva supernatant [14]. In Papua New Guinea, the sensitivity of RDT kit in detecting PfHRP2 in urine samples from malaria patients was reported to be 81.0% [15].

Even though previous studies identified PfHRP2 and pLDH in salivary and urine samples, detection of these proteins were done mostly with sensitive diagnostic techniques (enzyme immuno-assays and fluorescent immuno-assays), which are only available in reference and specialized laboratories. Malaria is mostly endemic in rural and peri-urban areas where there is lack of technical expertise to perform complex analytical assays and cost involved in establishing these assays is always high. Therefore, this study was designed to detect PfHRP2 and pLDH *P. falciparum* specific antigens in urine and salivary specimens from the same patients using readily available RDT kits and identify factors influencing the detection of these antigens in urine and saliva of *P. falciparum* infected individuals.

## Methods

### Study sites and period of sample collection

Blood, urine and saliva specimens were collected from clinically suspected malaria patients from Oduman Health Centre, Kotoku Health Centre, Mayera Health Centre and Pokuase Health Centre. These health centres are located in rural communities in the Ga West Municipality in the Greater Accra Region of Ghana. Samples were collected from October 2018 to May 2019.

### Included and excluded patients

Clinically suspected malaria patients included in this study were at least 5 years old. Patients less than 5 years and those unable to provide urine and saliva were excluded from the study.

### Sample size determination

Sample size was determined using the formula: n = z^2^p(1 − p)/d^2^, where n = sample size, p = prevalent of malaria in Ga West Municipality, *z* = confidence level at 95% (standard value of 1.96), d = margin of error at 5% (standard value of 0.05). The prevalence of malaria in Ga West Municipality was unknown so it was estimated at 50.0%. Therefore, the minimum sample size was calculated to be 384.

### Blood, urine and salivary samples collection

Blood samples (approx. 4 mL) were collected by venipuncture into K_3_EDTA tubes (Micropoint Diagnostics, China), 30 mL of urine and saliva (approx. 5 mL) were collected from the same patient. Due to difficulties in obtaining early morning urine, samples were collected between 6 a.m. and 10 a.m. Urine and salivary samples were frozen at − 20 °C till they were transported to Ga North Municipal Hospital Laboratory, Ofankor-Accra for analysis. However, whole blood was stored between 2 and 8 °C prior to analysis. Sample storage ranged between 15 and 48 h.

### Laboratory analyses

#### Malaria microscopy

Malaria parasites were quantified with the aid of Giemsa staining (pH = 6.8). Exactly, 6 µL of whole blood was used to prepare thick smears measuring approx. 2 cm in diameter. Air dried thick films were stained with 10% Giemsa for 10 min and asexual stages of parasites estimated using the WHO protocol for malaria parasites estimation [16]. In brief, parasite densities were determined by dividing the number of parasites counter per at least 200 leukocytes and multiplied by estimated WBC count of 8000 cells/µL.

#### Malaria rapid diagnostic testing using blood

Malaria parasites antigenaemia (PfHRP2 and pLDH) were detected by SD Bioline rapid diagnostic test kit (Gyeonggi-do, Republic of Korea) following manufacturer’s recommendation. In summary, 5 µL of well-mixed whole blood was dispensed into the sample window of the kit followed by four drops of buffer. Test results were read at 15 min.

#### Malaria rapid diagnostic testing using saliva and urine

PfHRP2 and pLDH antigens were detected in saliva and urine samples using SD Bioline rapid diagnostic test kit with some modifications. Optimized sample volumes were 20 µL of saliva and 30 µL of urine specimens. About 5 s after addition of samples into the sample window of the RDT kit, four drops of buffer were added. Test results were read at 15 min.

#### Determination of micro-haematuria and occult blood in saliva

Blood in saliva and urine specimens were detected using the blood determinant portion of Uritest 10E urine reagent strip (Guangxi, China) following the manufacturer’s procedure. Blood in the specimens were determined based on the pseudoperoxidase action of hemoglobin and erythrocytes which catalyzes the reaction 3,3′,5,5′-tetramethyl-benzidine and buffered organic peroxide. The blood determinant portion of the urine reagent strip was immersed in the sample (urine or saliva) for about 10 s. Results were read at 45 s. The resulting colors ranged from yellow-green to dark green depending on the amount of blood present in the sample. The test kit had sensitivity of 1 intact red blood cells/10 mL or 0.3–0.6 mg/L of haemoglobin.

#### Determination of haemoglobin concentrations

Haemoglobin concentrations were determined using Urit 5200 (Guangzhou, China) fully automated haematology analyzer. Well-mixed whole blood was aspirated by the analyzer and haemoglobin concentrations were determined automatically using cyanide free colorimetric method. Haemoglobin concentrations in g/dL were generated within a minute.

#### Data processing and statistical analysis

Laboratory data were processed by Microsoft Excel 2016. The sensitivities, specificities, positive and negative predictive values were determined based on the following formula and their respective 95% confidence intervals as well as the Chi-square goodness of fit of each technique were determined by SPSS Version 24 (Chicago, IL, USA).

$${\text{Sensitivity}} = {{{\text{true}}\;{\text{positive}}} \mathord{\left/ {\vphantom {{{\text{true}}\;{\text{positive}}} {\left( {{\text{true}}\;{\text{positive}} + {\text{false}}\;{\text{negative}}} \right)}}} \right. \kern-\nulldelimiterspace} {\left( {{\text{true}}\;{\text{positive}} + {\text{false}}\;{\text{negative}}} \right)}} \times 100\% ,$$

$${\text{Specificity}} = {{{\text{true}}\;{\text{negative}}} \mathord{\left/ {\vphantom {{{\text{true}}\;{\text{negative}}} {\left( {{\text{true}}\;{\text{negative}} + {\text{false}}\;{\text{positive}}} \right)}}} \right. \kern-\nulldelimiterspace} {\left( {{\text{true}}\;{\text{negative}} + {\text{false}}\;{\text{positive}}} \right)}} \times 100\% ,$$

$${\text{PPV}} = {{\text{true positive}} \mathord{\left/ {\vphantom {{\text{true positive}} {\left( {{\text{true positive}} + {\text{false positive}}} \right)}}} \right. \kern-\nulldelimiterspace} {\left( {{\text{true positive}} + {\text{false positive}}} \right)}} \times 100\% ,$$

$${\text{NPV}} = {{{\text{true}}\;{\text{negative}}} \mathord{\left/ {\vphantom {{{\text{true}}\;{\text{negative}}} {\left( {{\text{true}}\;{\text{negative}} + {\text{false}}\;{\text{negative}}} \right)}}} \right. \kern-\nulldelimiterspace} {\left( {{\text{true}}\;{\text{negative}} + {\text{false}}\;{\text{negative}}} \right)}} \times 100\% ,$$

where PPV and NPV are positive and negative predictive values respectively.

Accuracy was calculated based on the formula: Sensitivity × Prevalence + Specificity × (1 − Prevalence).

## Results

### Description of suspected malaria patients

A total of 864 suspected malaria patients were recruited for this study. Of this number 706 (81.7%) patients were able to provide all three samples (blood, urine and saliva) for analysis. Table 1 represents the demographic characteristics of suspected malaria patients that were able to provide all specimens. In all health facilities, more than 50% of the participants were females. The overall mean age (25th–75th percentile) of participants was 22.3 years (6–39 years). With the exception of participants recruited from Pokuase Health Centre, over 50% of all participants from other sites were less than 21 years.

**Table 1:** Demographic characteristics of suspected malaria patients

Demographic variables	Overall (n = 706)	Kotoku (n = 161)	Mayera (n = 107)	Oduman (n = 250)	Pokuase (n = 188)
Gender					
Females n (%)	405 (57.3%)	90 (55.9%)	71 (66.3%)	135 (54.0%)	108 (57.4%)
Males n (%)	301 (42.7%)	71 (44.1%)	36 (33.7%)	115 (46.0%)	80 (42.5%)
Age (years)					
1–10	225 (31.8%)	50 (31.0%)	19 (17.7%)	102 (40.8%)	54 (28.7%)
11–20	152 (21.5%)	36 (22.3%)	53 (49.5%)	43 (17.2%)	20 (10.6%)
21–30	107 (15.1%)	17 (10.5%)	17 (15.9%)	20 (8.0%)	53 (28.2%)
31–40	108 (15.3%)	27 (16.7%)	10 (9.3%)	41 (16.4%)	30 (15.9%)
41–50	72 (10.2%)	9 (5.6%)	8 (7.4%)	31 (12.4%)	24 (12.7%)
Above 50	42 (5.9%)	22 (13.6%)	0 (0.0%)	13 (5.2%)	7 (3.7%)

*Kotoku *Kotoku Health Centre, *Mayera *Mayera Health Centre, *Oduman *Oduman Health Centre, *Pokuase *Pokuase Health Centre

### Distribution of results by sample type and study sites

The overall prevalence of *P. falciparum* parasitaemia in Ga West Municipality by microscopy was 44.2% (312/706) while PfHRP2/pLDH antigenaemia was 53.9% (381/706). Highest malaria parasitaemia and antigenaemia was seen in Mayera Health Centre while relatively lower cases were seen in Pokuase Health Centre. Of the total malaria RDTs done on blood samples, 95% (362/381) were PfHRP2 while 5.0% (19/381) were both PfHRP2 and pLDH. Of the total of 706 urine samples tested, malaria antigens were detected in 134 samples (18.9%). Of the 134 positive urine samples, both PfHRP2 and pLDH antigens were detected in 15 (11.2%) samples while the rest contained only PfHRP2 antigens. In saliva samples, 217 out of 706 samples (30.7%) were found to contain malaria antigens. Both PfHRP2 and pLDH were detected in 92.2% (200/706) of saliva specimens while only PfHRP2 was detected in 7.8% (17/706) of saliva specimens. The distribution of the results in each study site is presented in Table 2.

**Table 2:** Sample related prevalence of *Plasmodium falciparum* antigens in the study sites

Sample type	Positive n (%)	Negative n (%)	Positive samples			
			Kotoku (n = 161) n (%)	Mayera (n = 107) n (%)	Oduman (n = 250) n (%)	Pokuase (n = 188) n (%)
Microscopy	312 (44.2)	394 (55.8)	69 (42.8)	67 (62.6)	103 (41.2)	73 (38.8)
Rapid diagnostic test (blood)						
Total PfHRP2/pLDH detected	381 (53.9)	325 (46.0)	94 (58.4)	71 (66.4)	127 (50.8)	89 (47.3)
Only PfHRP2 detected	362 [95.0]^a^		89 [94.7]^a^	67 [94.3]^a^	120 [94.4]^a^	86 [96.6]^a^
Both PfHRP2/pLDH detected	19 [5.0]^a^		5 [5.3]^a^	4 [5.6]^a^	7 [5.5]^a^	3 [3.7]^a^
Rapid diagnostic test (urine)						
Total PfHRP2/pLDH detected	134 (18.9)	572 (81.1)	27 (16.7)	31 (29.0)	39 (15.6)	37 (19.7)
Only PfHRP2 detected	119 [88.8]^a^		27 [100.0]^a^	27 [87.1]^a^	32 [82.0]^a^	33 [89.2]^a^
Both PfHRP2/pLDH detected	15 [11.2]^a^		0 [0.0]^a^	4 [12.9]^a^	7 [18.0]^a^	4 [10.8]^a^
Rapid diagnostic test (saliva)						
Total PfHRP2/pLDH detected	217 (30.7)	489 (69.3)	49 (30.4)	57 (53.3)	68 (27.2)	43 (22.9)
Only PfHRP2 detected	200 [92.2]^a^		45 [91.8]^a^	53 [92.9]^a^	63 [92.6]^a^	39 [90.7]^a^
Both PfHRP2/pLDH detected	17 [7.8]^a^		4 [8.1]^a^	4 [7.0]^a^	5 [7.3]^a^	4 [9.3]^a^

*Kotoku *Kotoku Health Centre, *Mayera *Mayera Health Centre,* Oduman *Oduman Health Centre,* Pokuase *Pokuase Health Centre

^a^Percentages*—*proportion of total RDT positives that were either PfHRP2 or pLDH positive

### Clinical characteristics of blood RDT confirmed malaria patients

In all, RDT detected PfHRP2/pLDH antigens in 132 (34.6%) patients with haemoglobin concentration less than 9.9 g/dL whilst in patients with haemoglobin concentration 10.0–12.5 g/dL and > 12.5 g/dL, PfHRP2/pLDH antigens were detected in 170 (44.6%) and 79 (20.7%) blood specimens. Over half of the salivary specimens were found to contain occult blood (52.2%), while only 29.4% of the urine samples were micro-haematuric. The overall mean temperature of the patients was 38.6 ± 0.72 °C. The distribution of the clinical findings among the four study sites are presented in Table 3.

**Table 3:** Clinical findings of patients with malaria parasites antigenaemia

Clinical parameters	Overall (n = 381)	Kotoku (n = 94)	Mayera (n = 71)	Oduman (n = 127)	Pokuase (n = 89)
Haemoglobin concentration					
< 9.9 g/dL n (%)	132 (34.6)	33 (35.1)	29 (40.8)	49 (38.5)	21 (23.6)
10.0–12.5 g/dL n (%)	170 (44.6)	43 (45.7)	31 (43.6)	57 (44.8)	39 (43.8)
> 12.5 g/dL n (%)	79 (20.7)	18 (19.1)	11 (15.4)	21 (16.5)	29 (32.6)
Occult blood in saliva					
Present n (%)	199 (52.2)	42 (44.7)	52 (73.2)	59 (46.4)	46 (51.6)
Absent n (%)	182 (47.8)	52 (55.3)	19 (26.8)	68 (53.5)	43 (48.3)
Micro-haematuria					
Present n (%)	112 (29.4)	29 (30.8)	27 (38.0)	31 (24.4)	25 (28.1)
Absent n (%)	269 (70.6)	65 (69.1)	44 (61.9)	96 (75.5)	64 (71.9)
Temperature (°C)					
37.5–38.0	78 (37.8)^a^	11 (11.7)^a^	7 (9.8)^a^	19 (14.9)^a^	10 (11.2)^a^
38.1–39.0	130 (38.7)^a^	14 (14.9)^a^	15 (21.1)^a^	33 (25.9)^a^	27 (30.3)^a^
> 39.0	173 (39.4)^a^	69 (73.4)^a^	49 (69.0)^a^	75 (59.0)^a^	52 (58.4)^a^

*Kotoku *Kotoku Health Centre, *Mayera *Mayera Health Centre,* Oduman *Oduman Health Centre,* Pokuase *Pokuase Health Centre

^a^Presented as number (mean)

### Sensitivities and specificities of urine and salivary specimens for detecting malaria antigens

Table 4 represents the diagnostic performance of urine and salivary specimens for diagnosing malaria. Compared to blood specimen, urine specimen was 35.2% sensitive while salivary specimen was 57.0% sensitive. However, both specimens had specificity and PPV of 100% although their NPVs differed. While urine sample was 65% accurate, saliva sample was 76.8% accurate. Compared to blood specimen, the detection of PfHRP2/pLDH antigens in both urine and salivary specimens were significantly different.

**Table 4:** Diagnostic performance of urine and saliva for detecting Plasmodium antigens with reference to blood

Diagnostic indices	Urine specimens	Saliva specimens
Overall indices of urine and saliva samples with blood contamination		
True positive	134	217
True negative	325	325
False positive	0	0
False negative	247	164
Sensitivity (95% CI)	35.2% (30.38–40.20)	57.0% (51.81–61.99)
Specificity (95% CI)	100% (98.87–100)	100% (98.87–100)
Accuracy (95% CI)	65.0% (61.37–68.53%)	76.8% (73.51–79.87%)
PPV (95% CI)	100.0	100.0
NPV (95% CI)	56.8% (55.00–58.62)	66.46% (63.84–68.99)
*χ*^2^ goodness of fit (p-value)	347.8 (< 0.05)	153.3 (< 0.05)
Diagnostic indices of urine and saliva samples without blood contamination		
True positive	22	28
True negative	325	325
False positive	0	0
False negative	269	182
Sensitivity (95% CI)	7.6% (4.8–11.2%)	13.3% (9.1–18.7%)
Specificity (95% CI)	100% (98.9–100)	100% (98.9–100)
Accuracy (95% CI)	50.2% (46.2–54.2)	53.3% (49.0–57.6)
PPV (95% CI)	100%	100%
NPV (95% CI)	48.1% (47.2–48.9)	49.7% (48.3–51.0)

*PPV* positive predictive value, *NPV* negative predictive value, *95% CI* 95% confidence interval

In urine samples without blood contamination, 22 samples were found to contain malaria antigens as well as their corresponding blood samples. As many as 325 urine and corresponding blood samples were found not to contain any of the malaria antigens. However, in 269 samples, no malaria antigens were detected while in their corresponding blood specimens, malaria antigens were detected. Based on the foregoing, without blood contamination, the sensitivity of urine samples to detect malaria antigens dropped to 7.6% while the sample remain very specific, with an accuracy of 50.2%. In salivary samples, using the figures presented in Table 4, the sensitivity was found to be higher than using urine samples but lower than previously determined sensitivity with blood contamination (13.3%) with an accuracy of 53.3%. Surprisingly, malaria antigens were detected in minimum parasitaemia of 63,150 parasites/µL and 57,335 parasites/µL from urine and saliva specimens respectively, although sample was not contaminated with blood.

### Factors influencing detection of malaria antigens in urine and saliva

In the 132 patients with haemoglobin concentrations less than 9.9 g/dL, malaria antigenuria was detected in 113 (85.6%) samples while 123 (93.1%) salivary samples were found to contain malaria antigens. As haemoglobin concentration increases, the number of urine and salivary samples found to contain malaria antigens drastically reduces. Rate of detection of malaria antigenuria (R = 0.91, p = 0.004) and salivary malaria antigen (R = 0.95, p = 0.001) increased with increasing malaria parasitaemia. It is noteworthy that malaria antigenuria was not detected in patients with parasite count less than 60,000 parasites/µL of blood. But in the case of salivary specimens, parasitaemia was detected when parasitaemia was above 40,000 parasites/µL (Table 5).

**Table 5:** Levels of haemoglobin concentration and parasite count influencing detection of parasites antigens in urine and saliva

Parameter	Number of respective samples found to contain PfHRP2 and/or pLDH			
	Blood	Urine	Saliva	*χ*^2^ (p-value)
Haemoglobin concentration				29.5 (p < 0.05)
< 9.9 g/dL n (%)	132	113 (85.6)	123 (93.1)	
10.0–12.5 g/dL n (%)	170	15 (8.8)	77 (45.2)	
> 12.5 g/dL n (%)	79	6 (7.5)	17 (21.5)	
Parasite count (/µL of blood)				14.7 (p < 0.05)
20,001–40,000	51	0 (0.0)^a^	0 (0.0)^a^	
40,001–60,000	46	0 (0.0)^a^	17 (36.9)^a^	
60,001–80,000	57	9 (15.7)	9 (15.7)	
80,001–100,000	67	13 (19.4)	51 (76.1)	
100,001–120,000	82	48 (58.5)	71 (86.5)	
> 120,000	78	64 (82.0)	69 (88.5)	

^a^Excluded from Chi-square analysis. Percentages indicated for urine and saliva section represent the proportions of Plasmodium antigens in respective specimens with respect to antigens identified in blood specimens for a particular parameter

In 199 salivary specimens with occult blood, PfHRP2/pLDH antigens were detected in 95.0% (189/199) of them whereas 28 out of 182 salivary samples (15.4%) without occult blood were found to contain malaria antigens. However, malaria antigens were detected urine of all malaria patients exhibiting haematuria while in the 269 samples without haematuria, 22 (8.2%) were found to contain malaria antigens. It was also observed that as body temperature increased the rate of detection of PfHRP2/pLDH antigens in urine and salivary specimens also increased (Table 6).

**Table 6:** Factors affecting release of parasite antigens into urine and saliva

Sample type	Urine specimen		Saliva specimen	
PfHRP2/pLDH	Positive n (%)	Negative n (%)	Positive n (%)	Negative n (%)
Occult blood in saliva				
Present n (%)	NA	NA	189 (95.0)	10 (5.0)
Absent n (%)	NA	NA	28 (15.4)	154 (84.6)
Micro-haematuria				
Present n (%)	112 (100.0)	0 (0.0)	NA	NA
Absent n (%)	22 (8.2)	247 (91.8)	NA	NA
Temperature (°C)				
37.5–38.0 n (%)	7 (8.9)	71 (91.0)	15 (19.2)	63 (80.7)
38.1–39.0 n (%)	13 (10.0)	117 (90.0)	46 (35.4)	84 (64.6)
> 39.0 n (%)	114 (65.9)	59 (34.1)	156 (90.2)	17 (9.8)

*NA *not applicable

## Discussion

The use of urine and saliva as a non-invasive procedure to detect malaria parasites that avoid the use of blood with no need of special equipment and may be suitable for societies with blood taboos [12, 17]. In surveillance studies, blood sampling and in some cases repeated blood sampling often dissuade many study subjects from involving themselves in clinical and epidemiological studies [18, 19]. Research findings also confirm that patients are usually willing to donate any other samples than blood towards biomedical studies [20]. Based on these, if analytical procedures are modified to use other human samples instead of blood and other invasive procedures, patronage of biomedical studies will increase with its attendant benefits of reduction in transmission of blood borne infections.

Previous studies attempted to evaluate the use of non-blood samples to detect malaria antigens. In India, the sensitivities of urine in two different studies were found to be 38% [21] and 86.67% [22]. Like this study, rapid diagnostic test kits intended to be used for detecting malaria antigens in blood were used. In another studies, diagnostic kits specifically meant for detecting malaria antigens in urine were used. Oyigbo et al. [23] reported sensitivity of 85% while Oguonu et al. [24] also reported sensitivity of 84%. However, these previous studies failed to report the conditions under which malaria antigens were detected in non-blood samples. Urine has been widely evaluated as an alternative specimen for malaria diagnosis. Few studies have evaluated the use of saliva. In this study, saliva, with or without blood contamination, was found to be of higher sensitivity compared to urine as an alternative sample to diagnose malaria. This study has provided some conditions that could enhance the detection of malaria antigens in urine and saliva. The identified conditions were sample contamination with blood, malaria parasitaemia > 60,000 parasites/μL, moderate to severe anaemia and hyperthermia (temperature > 38.7 °C). Considering the low sensitivities observed in this study compared to previous studies, it could be due to lower concentrations of the antigens in random samples and/or degradation of malaria antigens in urine samples from time of collection to analysis of specimens [11].

In spite of these limitations, higher detection rate of *P. falciparum* specific antigens was observed in saliva compared to urine. This could probably be because, in uncomplicated malaria where fever is characterized by body temperatures above 37.5 °C, vasodilation of vessels supplying the buccal cavity, the skin and other body openings is a possibility. This will ensure the capillaries underneath the skin and other mucous membranes are filled with blood if the body gets too hot. Subsequently, blood is brought closer to surfaces so more heat can be lost. Moreover, gingivitis is common in malaria so with the least trauma to the gum, blood is released into the mouth which can contaminate saliva for subsequent detection of *P. falciparum* specific antigens. This study reports 57.0% detection rate of *P. falciparum* specific antigens in saliva whilst previously *P. falciparum* DNA has been detected in human saliva (73%) [11, 25]. Again similar to this study, pLDH was detected in saliva in previous study in Nigeria. That study reported 77.9% sensitivity of detected pLDH in saliva [14] while in this study, the detection rate of pLDH was 89.4% (17/19). Again, *P. falciparum* HRP2 antigens were detected in saliva of malaria patients using ELISA technique, and even though the sensitivity was low (43%) with comparatively long turnaround time (approx. 2 h and 15 min) [12], this report together with findings in this study showed the potential of a non-invasive approach for malaria diagnosis using saliva. High detection rate of pLDH confirms the usefulness of saliva in detecting active malaria where concentration of pLDH is very high and short-lived. The presence of pLDH has been associated with malaria parasite viability in some studies [26, 27].

Again, in this study, urine samples of 35.2% of acute malaria patients were found to contain PfHRP2 and pLDH proteins. It has been previously reported that kidney involvement is not uncommon in falciparum and malariae malaria [28]. Haemodynamic dysfunction and immune response are the main mechanism of malaria associated kidney pathology [29]. Furthermore, malaria has been reported as the first parasitic infection to be clearly associated with glomerular diseases in tropical areas [30] with subsequent detection of *Plasmodium* antigens in the glomeruli [28]. Proteinuria and microalbuminuria associated with kidney involvement in malaria [31] made it possible for malaria specific proteins to be detected in urine, as was observed in this study. The molecular weight of albumin is 69 kDa [32] whilst that of PfHRP2 is 30 kDa [33], and pLDH is 32 kDa [34]. Thus, the respective molecular weight of PfHRP2 and pLDH is less than half the molecular weight of albumin, so if albumin is excreted in urine in uncomplicated malaria, then PfHRP2 and pLDH could be freely excreted as well.

In *P. falciparum* infected patients, over 85% of salivary and urinary PfHRP2 and pLDH were identified in haemoglobin concentration less than 9.9 g/dL, body temperature greater than 38.1 °C, occult blood in saliva and urine and parasitaemia of > 60,000 parasites/µL of blood.

*Plasmodium falciparum* is an obligatory intraerythrocytic parasite so destruction of red cells containing parasites and uninfected red cells resulting in anaemia is very common [35]. In severe malaria, hemoglobin (molecular weight = 65 kDa) [36] in urine has been reported as well as nephrotic syndrome especially in *P. malariae* infections [37]. Based on these separate reports, association of malaria antigenuria with anaemia is not surprising. Moreover, the smaller molecular sizes of these malaria antigens and their water solubility make them easily excreted in urine, hence their detection. Additionally, anaemia in malaria is further worsened by discharge of blood in body fluids. Malaria antigens were detected in all haematuric infected patients, and in 95% of malaria patients with positive occult blood in saliva. Meanwhile, PfHRP2 proteins are found on the surface of infected red blood cells [33], so once red cells are detected in urine and saliva, detection malaria antigens in these specimens is highly possible.

It has been reported that glomerular capillary dilatation, haemorrhage into the interstitium, in small and large renal vessels occur during pathogenesis of fever [38], also acute gingival bleeding has been reported in fever especially in dengue hemorrhagic fever [39]. Vasodilation of the renal vasculature and gingival bleeding as a result of hyperthermia enhanced the detection of malaria antigens in urine and saliva in this study.

Finally, even though detection of malaria antigens improved with increased parasitaemia, Gbotosho et al. [14] found otherwise. In their study, RDT failed to detect parasite antigen in some saliva samples, despite high parasitaemia (2,571–334,298 parasite/µL blood) and positive RDT in matching whole-blood samples from the same patients. The reasons for this disparity could be due to the difference in the sensitivities of the malaria RDT used in these two studies (SD Bioline was used in this study, while Gbotosho et al. [14] used OptiMAL-IT dipstick). Again, in their study, patients with concomitant illness were excluded while in this study, patients with gingivitis (evidenced by occult blood in saliva) and haematuria were included. Also, this study did not exclude any patient with possible concomitant infections (other infectious disease markers were not screened). So the differences in the study patients could also account for the differences observed with respect to the detection threshold of parasitaemia that influenced detection of malaria antigens in saliva. In addition, Gbotosho et al. [14] performed RDT strictly according to manufacturer’s instruction as validated for blood sample testing while in this study, the detection of malaria proteins was optimized to increase improve sensitivity.

## Conclusion

The overall prevalence of malaria detectable by microscopy and RDT in Ga West Municipality was 44.2% and 53.9% respectively. Of the total patients found to be positive by RDT, 35.2% and 57.0% had malaria antigens detected in their urine and saliva respectively; confirming the possibility of detecting *P. falciparum* specific antigens in urine and saliva. Sensitivity of detecting malaria antigens in these specimens improves in severe to moderate anaemia, hyperthermia, parasitaemia > 60,000 parasites/µL of blood and occult blood in urine and saliva. Available RDT kits were validated for blood samples where malaria antigens concentrations are very high. It is recommended that RDT kits be developed for other body fluids where lower amount of malaria antigens could be present and may degrade faster.

## Limitations

Study design did not exclude individuals with other co-morbidities that could enhance or reduce the detecting of malaria antigens in urine and saliva. Also, determination of PfHRP2 antigen levels in these alternative samples was not done.

## Data Availability

All data generated or analysed during this study are included in this published article.

## Abbreviations

GWM: Ga West Municipality
KHC: Kotoku Health Centre
MHC: Mayera Health Centre
OHC: Oduman Health Centre
PfHRP2: *Plasmodium falciparum* HISTIDINE-rich protein 2
PHC: Pokuase Health Centre
pLDH: *Plasmodium* Lactate dehydrogenase
RDT: Rapid diagnostic test kits
SSA: Sub-Saharan Africa
